# Characterization and functional analysis of the adipose tissue-derived stromal vascular fraction of pediatric patients with osteogenesis imperfecta

**DOI:** 10.1038/s41598-022-06063-4

**Published:** 2022-02-14

**Authors:** Josephine T. Tauer, Hadil Al-Jallad, Mayumi Umebayashi, Dena Bakhsh, Damian Rauch, Simon D. Tran, Frank Rauch, Reggie Hamdy

**Affiliations:** 1 Faculty of Dental Medicine and Oral Health Sciences, McGill University, 2001 Avenue McGill College, Montréal, QC H3A 1G1 Canada; 2 Shriners Hospital for Children-Canada, 1003 Decarie Blvd, Montreal, QC H4A 0A9 Canada; 3 Faculty of Medicine, McGill University, 3605 Rue de la Montagne, Montréal, QC H3G 2M1 Canada; 4 Department of Pediatrics, Faculty of Medicine, McGill University, 1001 Decarie Boulevard, Montreal, QC H4A 3J1 Canada; 5 Experimental Surgery, McGill University, 1650 Cedar Avenue, Montreal, QC H3G 1A4 Canada

**Keywords:** Mesenchymal stem cells, Regeneration, Stem-cell research, Paediatric research

## Abstract

Pediatric patients with Osteogenesis Imperfecta (OI), a heritable connective tissue disorder, frequently suffer from long bone deformations. Surgical correction often results in bone non-unions, necessitating revision surgery with autogenous bone grafting using bone-marrow-derived stem cells (BM-SC) to regenerate bone. BM-SC harvest is generally invasive and limited in supply; thus, adipose tissue's stromal vascular fraction (SVF) has been introduced as an alternative stem cell reservoir. To elucidate if OI patients' surgical site dissected adipose tissue could be used as autologous bone graft in future, we investigated whether the underlying genetic condition alters SVF's cell populations and in vitro differentiation capacity. After optimizing SVF isolation, we demonstrate successful isolation of SVF of pediatric OI patients and non-OI controls. The number of viable cells was comparable between OI and controls, with about 450,000 per gram tissue. Age, sex, type of OI, disease-causing collagen mutation, or anatomical site of harvest did not affect cell outcome. Further, SVF-containing cell populations were similar between OI and controls, and all isolated SVF's demonstrated chondrogenic, adipogenic, and osteogenic differentiation capacity in vitro. These results indicate that SVF from pediatric OI patients could be used as a source of stem cells for autologous stem cell therapy in OI.

## Introduction

Osteogenesis imperfecta (OI), a rare hereditary connective tissue disorder, is usually caused either by a dominant mutation in one of the collagen type I coding genes, *COL1A1* or *COL1A2, *or by recessive mutations in other genes, such as *SERPINF1, CRTAP, WNT1*, involved in collagen type I biosynthesis, function, transport, or secretion^1,2^. Based on the clinical severity, OI is classified into 4 groups: type I 'non-deforming OI', type II 'perinatally lethal', type III 'progressively bone deforming OI', and type IV 'moderate to severe form of OI'^3,4^. Further types of OI have been described based on their clinical manifestation and underlying genetic mutation (OI type V and higher)^4^.

So far, there is no cure for OI yet. Therefore, the overall goal of OI therapy is to optimize the patient’s gross motor abilities and maximize independence. This is largely accomplished by treatment with anti-resorptive drugs, by physical rehabilitation, and orthopedic interventions to stabilize fractures and correct spinal and long bone deformities^2^.

Today it is generally accepted that the best treatment for long bone deformities is by correcting the alignment of the long bones with osteotomies and intra-medullary rodding. This surgical technique usually yields satisfactory results with improvement in ambulation, daily activities, and quality of life. But, one of the side effects of this surgery is delayed union or non-union at the osteotomy site, which can lead to pain, bending of the intra-medullary rod, repeated fracture, and loss of ambulation^5–7^. Additional surgical intervention may be required that often involves revision of intramedullary fixation and bone grafting. Although autogenous bone grafting (ABG) is still considered the gold standard, ABG has several drawbacks including limited graft supply, specifically in young children; invasive surgical procedures to harvest the bone graft; and potential complications at the donor site, most commonly persistent pain.

During fracture repair, mesenchymal stem cells (MSC) play a key role and, therefore, have been of main interest for bone tissue engineering and regenerative medicine. Sources of mesenchymal stem cells (MSC) include numerous tissues such as bone marrow (BM), synovial fluid, amniotic fluid, amniotic membrane, dental tissues, endometrium, limb bud, menstrual blood, peripheral blood, placenta and fetal membrane, salivary gland, skin and foreskin, sub-amniotic umbilical cord lining membrane, and Wharton's jelly^8^. BM is the most used source for MSCs, but, unfortunately, BM aspirates are not consistent or sufficiently rich in MSCs and represent a highly invasive procedure. Further, BM-MSCs lose their ability to proliferate and differentiate with increasing risk of cell senescence^9^. As alternative, it has been shown that the stromal vascular fraction (SVF) of adipose tissue contains cells that express specific stem cell markers and have similar differentiation capacity as BM-MSCs^10,11^. The main advantage of adipose tissue-derived stem cells (ASCs) is the accessibility of such cells and simpler isolation procedure^12^. Further, one gram of fat has 1,000 times more stem cells than one gram of BM^13^. Additionally, equivalent to BM-MSCs, ASCs were shown to have anti-inflammatory, angiogenic, immunomodulatory, and regenerative properties^14^. Further, stem cell quality and proliferation capacity do not decline with patient age^15,16^.

Hence, SVF of adipose tissue seems to be a promising source of ASCs that could be used as an autologous bone graft to prevent non-unions in OI. Harvesting adipose tissue from the surgical site of an osteotomy would permit a clinical "one-step-approach" of adipose tissue harvest, SVF preparation, and re-implantation in the patient's surgical site within the same surgical procedure to prevent a non-union of the corrected bone in the same patient. In this way, the patient's SVF would serve as an autologous tissue/cell/bone graft. This strategy would minimize the need for additional surgeries to correct non-unions, reduce pain, and improve the life quality of pediatric OI patients. Further, SVF preparation carried out in the operating room parallel to the surgical procedure would avoid taking the adipose tissue outside the operating room and culturing before re-implantation and avoid the numerous regulatory hurdles imposed by the FDA and Health Canada on allogeneic cell transplants. But, as OI is a systemic disorder affecting many connective tissues, it is presently unknown whether the cell populations of adipose tissue are altered in OI and whether SVF containing ASCs obtained from OI patients have multi-differentiation capacity. Further, available protocols for human SVF isolation uses lipoaspirates^10,17–22^; accordingly, it remains unclear if SVF isolation method can be used for resected adipose tissue (from the surgical site) of OI patients and if this method can be performed in a time span of a surgical procedure.

Accordingly, this study aimed to evaluate if (a) isolation of ASC-containing SVF is feasible in patients with OI compared to non-OI controls, (b) that it is possible to isolate ASC-containing SVF from both sexes and young patients, (c) if SVF cell subpopulation is altered in OI, (d) that the isolation is achievable within 90 min, and (e) that isolated ASCs display in vitro multi-differentiation capacity into osteogenic, adipogenic, and chondrogenic lineage.

## Results

### Patient characteristics

Between August 2017 and November 2018, adipose tissue samples were harvested from 40 patients (age range: 2–22 years; 19 males, 21 females) during corrective orthopedic surgery. Thirty-six samples were harvested from patients diagnosed with OI (OI type I, n = 1; OI type III, n = 10; OI type IV, n = 19; OI type V, n = 2; OI type VI, n = 1;OI type VII, n = 3) (age range: 2–22 years; 18 males, 18 females) and four samples from healthy controls (HC, age range: 4–14 years; 1 male, 3 females; no diagnosis of genetic musculoskeletal diseases). Demographics of OI patients and HCs are shown in Supplemental Table A.1.

### Isolation and cellular outcome of the stromal vascular fraction from pediatric adipose tissue

The overall mean weight of harvested adipose tissue was 6.86 g (SEM: 1.17 g) in the OI group and 7.48 g (SEM: 3.82 g) in controls. Within about 90 min, adipose tissue samples were processed to obtain SVF from HC and OI donors. All 40 SVF isolations were successful. The overall average number of nucleated cells per gram of adipose tissue was 443,000 cells per gram tissue (SEM: 6.66 × 10^4^ cells/g) in OI and 462,000 cells per gram tissue (SEM: 17.13 × 10^4^ cells/g) in HC (Table 1). Cell outcome did not vary significantly with sex, age, type of OI, disease-causing collagen mutation, or anatomical site of harvest (Supplemental Table A.2).

**Table 1:** Characteristics of isolated adipose tissue samples.

	HC patients (N = 4)	OI patients (N = 38)	P–value
Adipose tissue weight (g)	7.48 ± 3.82 (2.50–18.8)	6.86 ± 1.17 (1.2–36.0)	0.84
Number of cells per gram of adipose tissue (× 10^4^)	46.25 ± 17.13 (19.0–96.0)	44.37 ± 6.66 (1.25–175)	0.59
Total viable cells yield (× 10^6^)	2.31 ± 0.94 (0.75–5.0)	2.78 ± 0.39 (0.25–10)	0.80

Data represent mean ± SEM (minimum − maximum). P values represent significance of the difference between HC and OI patients using Mann–Whitney-U-Test.

#### Quantification of cell subpopulation of the stromal vascular fraction

**Figure 1:**
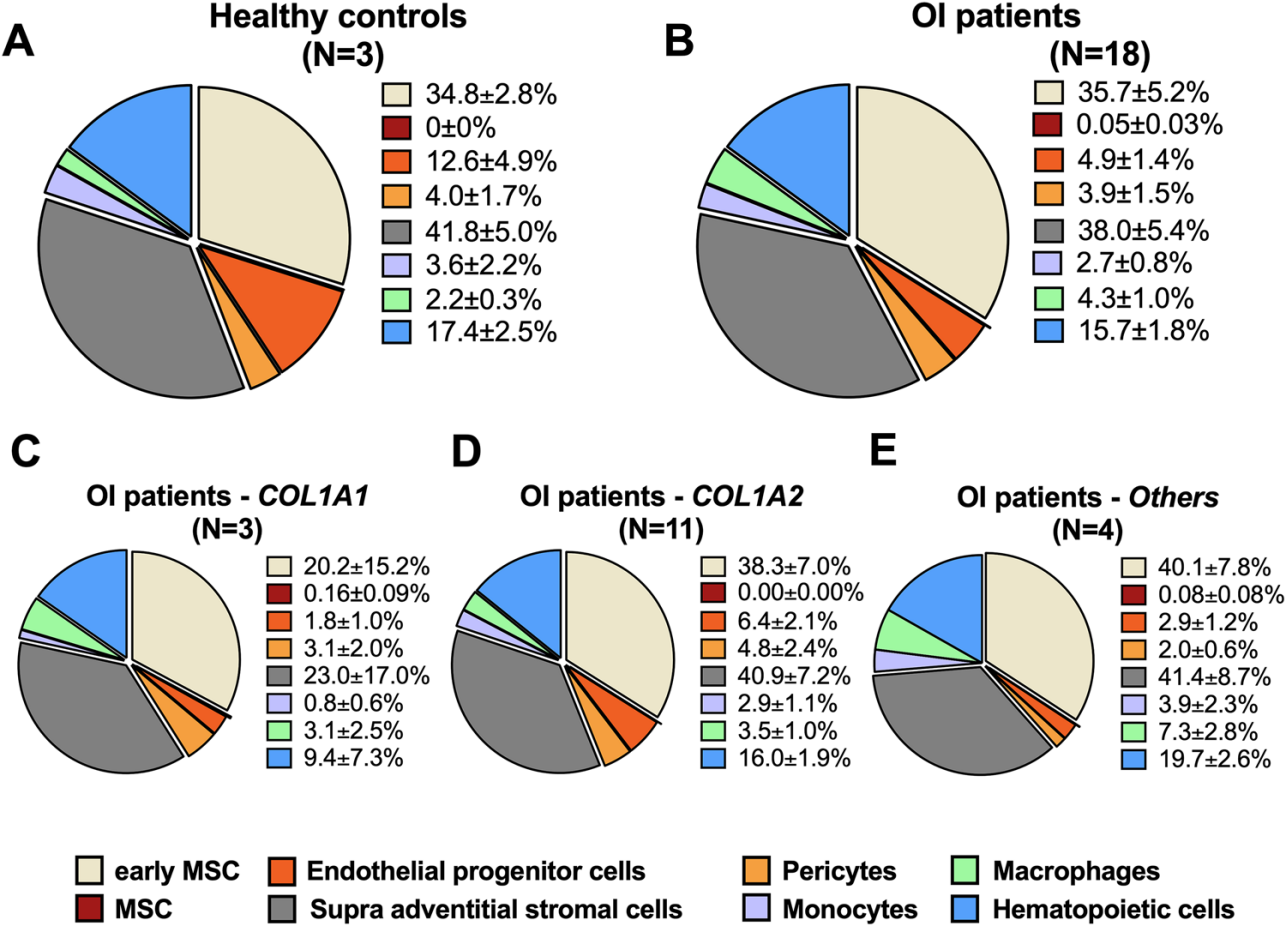
Quantified cellular subpopulations of the stromal vascular fraction of healthy controls (**A**) and OI patients (**B**). (**C**–**E**) Depicts cellular subpopulations of the stromal vascular fraction of OI patients according to genetic mutation. Others: OI patients with mutation in either *SERPINF1*, *CRTAP*, *IFITM5* and *WNT1*. Values represent mean ± SEM. MSC, mesenchymal stem cells.

Distribution analysis of SVF cell populations revealed similar number of viable cells in OI and HCs (Table 2). However, significantly lower endothelial progenitor cells (CD45^-^/CD34^+^/CD31^+^) were found in OI than in HC samples (p = 0.035), while early ASCs (CD45^-^/CD34^+^/CD73^+^/CD90^+^), hematopoietic cells (CD45^+^), monocytes (CD45^+^/CD14^+^/CD206^-^), macrophages (CD45^+^/CD14^+^/CD206^+^), and pericytes (CD45^-^/CD34^-^/CD146^+^) were similar between HC and OI patients (Table 2, Fig. 1).

**Table 2:** Composition of the stromal vascular fraction.

	HC patients (N = 3)	OI patients (N = 18)	P–value
Viability	80.5 ± 5.3% (71.8–90.2)	85.0 ± 4.0% (44.3–97.6)	0.31
Early mesenchymal stem cells	34.8 ± 2.8% (30.0–39.9)	35.7 ± 5.2% (2.2–85.5)	0.95
Activated mesenchymal stem cells	0 ± 0% (0–0)	0.05 ± 0.03% (0–0.33)	0.46
Endothelial progenitors	12.6 ± 4.9% (4.4–21.4)	4.9 ± 1.4% (0.4–20.3)	**0.035**
Pericytes	4.0 ± 1.7% (1.2–6.9)	3.7 ± 1.4% (0.1–24.7)	0.37
Supra-adventitial stromal cells	41.8 ± 5.0% (32.7–49.9)	38.0 ± 5.5% (2.6–87.2)	0.79
Monocytes	3.6 ± 2.2% (0.3–7.9)	2.7 ± 0.8% (0.04–11.4)	0.55
Macrophages	2.2 ± 0.3% (1.7–2.8)	4.3 ± 1.0% (0.3–13.4)	0.69
Hematopoietic cells	17.4 ± 2.5% (12.8–21.5)	15.8 ± 1.8% (0.6–29.3)	0.72

Data represent mean ± SEM (minimum—maximum). P values represent significance of the difference between HC and OI patients using Mann–Whitney-U-Test.

Statistical evaluation of the effect of age, sex, type of OI, disease-causing collagen mutation, and anatomical side of harvest on cell viability and cellular composition revealed i) significantly higher cell viability in SVF from OI type III donors (p = 0.011) while number of endothelial progenitor cells were found higher in OI type IV donors (p = 0.048); ii) higher amount of early ASCs (p = 0.02), supra-adventitial stromal cells (p = 0.02), endothelial progenitor cells (p = 0.007), and monocytes (p = 0.04) in SVF from male donors; and iii) higher amount of early ASCs (p = 0.015) and supra-adventitial stromal cells (p = 0.023) in SVF from postpubertal (age > 16 years) donors while pericytes were found higher in pre- and postpubertal donors (p = 0.014, Supplemental Tables A.3 and A.4). Though, the anatomical site of harvest had no effect on cell viability and cellular composition (Supplemental Tables A.3 and A.4).

### HC- and OI- ASC reveal similar multi-lineage differentiation capacity

**Figure 2:**
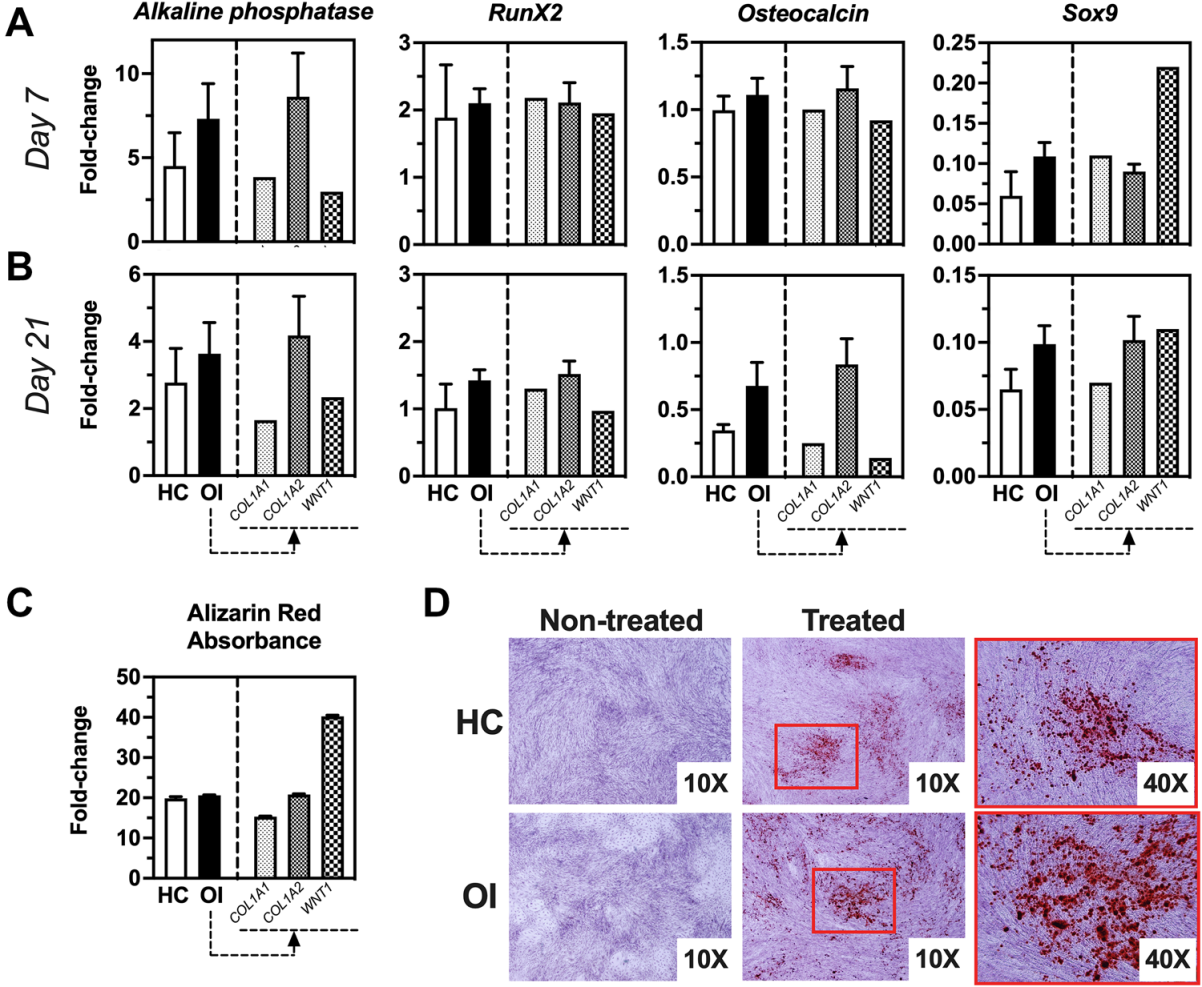
Osteogenic gene expression profile of SVF cells obtained from healthy controls (HC) or OI patients at days 7 and 21 in culture. (**A**) Fold-change of gene expression of treated versus non-treated cells at day 7 of culture. (**B**) Fold-change of gene expression of treated versus non-treated cells at day 21 of culture. (**C**) Quantification of alizarin red staining via absorbance measurements displayed as fold-change of treated versus non-treated samples. (**D**) Representative images of alizarin red staining of treated and non-treated HC and OI samples. HC, n = 2; OI, n = 9; OI-*COL1A1*, n = 1; OI-*COL1A2*, n = 6; OI-*WNT1*, n = 1. *ALP* alkaline phosphatase; *OCN* osteocalcin. Unpaired t-test of genetic fold-change of HC versus OI patients showed similar expression for all genes tested.

**Figure 3:**
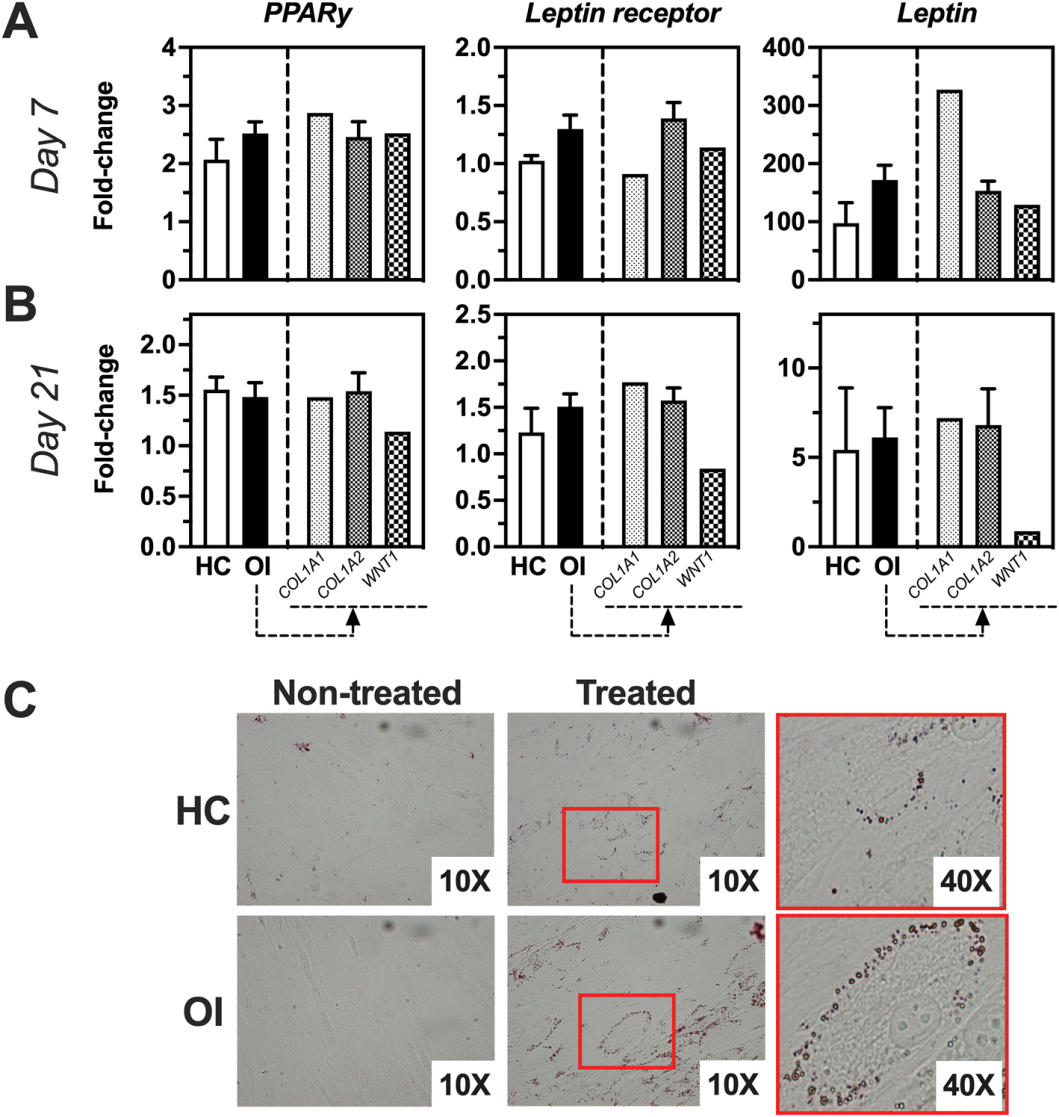
Adipogenic gene expression profile of SVF cells obtained from healthy controls (HC) or OI patients at days 7 and 21 in culture. (**A**) Fold-change of gene expression of treated versus non-treated cells at day 7 of culture. (**B**) Fold-change of gene expression of treated versus non-treated cells at day 21 of culture. (**C**) Representative images of oil red staining of treated and non-treated HC and OI samples. HC, n = 2; OI, n = 9; OI-*COL1A1*, n = 1; OI-*COL1A2*, n = 6; OI-*WNT1*, n = 1. Unpaired t-test of genetic fold change of HC versus OI patients showed similar expression for all genes tested.

**Figure 4:**
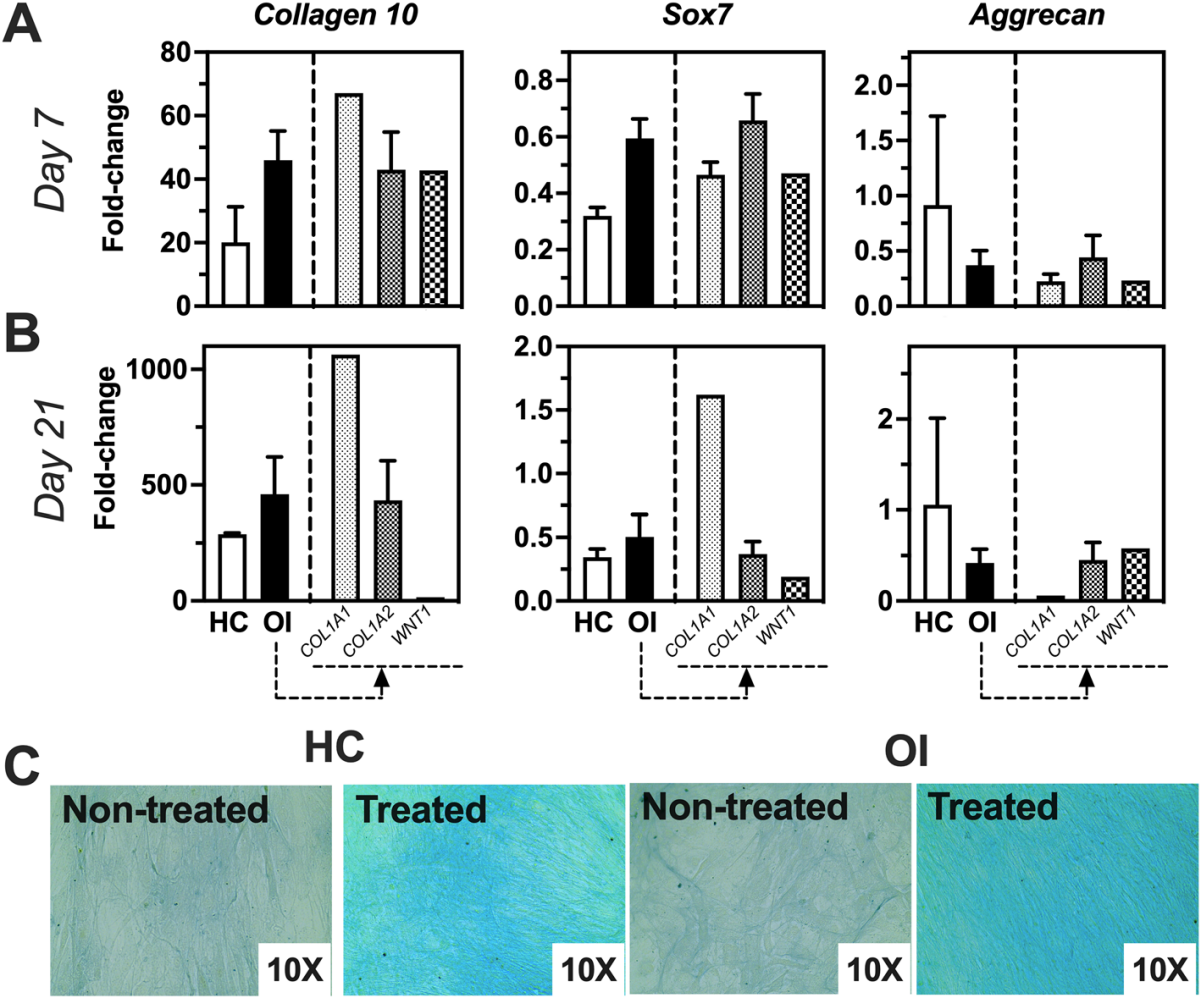
Chondrogenic gene expression profile of SVF Cells obtained from healthy Controls (HC) or OI patients at Days 7 and 21 in culture. (**A**) Fold-change of gene expression of treated versus non-treated cells at day 7 of culture. (**B**) Fold-change of gene expression of treated versus non-treated cells at day 21 of culture. (**C**) Representative images of alcian blue staining of treated and non-treated HC and OI samples. HC, n = 2; OI, n = 9; OI-*COL1A1*, n = 1; OI-*COL1A2*, n = 6; OI-*WNT1*, n = 1. Unpaired t-test of genetic fold-change of HC versus all OI patients showed similar expression for all genes tested.

When SVF cells were cultured under osteogenic conditions, expression of *Alkaline Phosphatase (AlkP)*, *Runx2*, *Sox9*, and *Osteocalcin (OCN)* at day 7 and day 21 was higher than in non-treated samples. HC and OI samples showed similar results (Fig. 2A,B). Alizarin red staining of deposited minerals at day 21 confirmed successful differentiation towards functional osteoblasts (Fig. 2C,D)*.* When SVF cells were cultured under adipogenic conditions, *PPARy*, *Leptin receptor* (*LEPR)*, and *Leptin* were significantly upregulated and revealed similar results for HC and OI samples (Fig. 3A,B). At day 21, oil droplet accumulation confirmed adipogenic differentiation (Fig. 3C). Under chondrogenic conditions, we observed significant upregulation of *Collagen 10*, *Sox7*, and *Aggrecan* (*ACAN*) with comparable results for HC and OI cells (Fig. 4A,B). Alcian blue staining confirmed chondrogenic differentiation (Fig. 4C).

Evaluation of the influence of collagen mutation on multi-lineage differentiation capacity revealed similar differentiation competence towards osteogenic and adipogenic lineage of SVF cells with *COL1A1*, *COL1A2*, and *WNT1* mutations. Regarding chondrogenic lineage, SVF cells harboring *COL1A1* mutations seemed to have a higher differentiation competence than mutations in other genes. But, due to the heterogenous sample distribution (OI-*COL1A1*, n = 1; OI-*COL1A2*, n = 6; OI-*WNT1*, n = 1), a conclusion cannot be drawn yet.

## Discussion

In this study, we successfully demonstrated the isolation of SVF from resected adipose tissue of pediatric patients with OI and HC within about 90 min. The number of isolated cells and cell viability was comparable between OI and HC and independent of age, sex, anatomical site of harvest, or genetic OI mutation. Additionally, SVF obtained from OI and HC revealed similar amount of stem cells and tri-lineage differentiation capacity in vitro.

To isolate SVF containing ASCs, traditionally harvested lipoaspirate is exposed to enzymatic dissociation followed by several centrifugation steps^10,18–22^. This is a relative time-consuming procedure and could not be performed in OI if isolated SVF is immediately be used for induction of bone regeneration within the same bone-corrective surgery. Therefore, we performed SVF isolation according to the technique described by Tevlin et al.^17^ with minor modifications in order to use this method for resected adipose tissue and optimized processing time. With this protocol, we were able to isolate SVF with about 90% viable cells from OI and HC. This yield is comparable to established non-intraoperative isolation protocols and intraoperative isolation procedures applied to adipose tissue or lipoaspirates^23^. Further, isolated ASCs presented the typical stem cell markers and quantity of ASCs isolated from dissected adipose tissue comparable to previously published yields achieved from pediatric and adult patients^24^ and by liposuction and enzymatic ASC isolation (about 25%–30%)^21^. Additionally, we found that the yield of ASCs was irrespective of anatomical harvest site as described before^25,26^.

Yet, the stromal cell population including pericytes, ASCs, and supra-adventitial stromal cells, are the most important cell types in regenerative therapies because of their multi-lineage differentiation capacity^15,27^. Supra-adventitial stromal cells and pericytes are both identified as precursor cells of ASCs, although there still remains some discussion^28–31^. Composition of isolated SVF from OI and HC was similar to each other and comparable to published data^23^. Still, SVF from OI patients revealed a significantly lower percentage of endothelial progenitor cells, which play a role in angiogenesis^32^. Angiogenesis is a key factor in bone repair as new blood vessels bring oxygen and nutrients to the highly metabolically active regenerating callus and serve as a route for inflammatory cells, cartilage, and bone precursor cells to reach the injury site^33^. The lower percentage of endothelial progenitor cells in OI suggests a diminished capacity of angiogenesis in OI and remains a question for future studies.

Regarding multi-lineage differentiation capacity, ASCs from OI and HC had similar osteogenic, adipogenesis, and chondrogenic differentiation capacity and are in line with published studies of human ASCs^10,15,34–37^. Concerning the underlying collagen mutation, we found similar differentiation capacity towards osteogenic and adipogenic lineage. ASCs derived from OI patients with *COL1A1* mutation seemed to have higher chondrogenic differentiation capacity suggesting that they are more susceptible to TGF-β induced chondrogenesis. TGF-β is known to stimulate chondrogenic differentiation^38^. Further, TGF-β seems to play a role in OI pathology as mouse models of recessive (mutation in the *Crtap* gene; *Crtap*^−/−^) and dominant (collagen type I mutations; *Col1a2*^*tm1.1Mcbr*^ & *Col1a1*^*Jrt/*+^) OI showed excessive TGFβ-signaling in the skeleton^39,40^. Interestingly, anti-TGFβ treatment using a neutralizing antibody corrected bone fragility in *Crtap*^−/−^ and *Col1a2*^*tm1.1Mcbr*^ mouse model but not in the *Col1a1*^*Jrt/*+^ mouse model, suggesting a link between collagen mutation and TGF-β signaling^39,40^. However, our sample size was too small to draw definite conclusions, and further studies are needed.

A limitation of our study is that we investigated pediatric patients only as it has been shown before that ASC isolation and bone regeneration/wound healing of autologous transplanted ASCs in patients between 6 and 72 years of age were similar^24,41,42^. Further, we did not compare multi-lineage differentiation of isolated ASCs to BM-MSCs as it also has been demonstrated before that compared to BM-MSCs, ASCs have a better resistance to cell senescence^43,44^ and are more effective in multi-lineage differentiation^44–48^. Additionally, we did not investigate cell senescence as it was demonstrated before that ASCs cell characteristics are stable for up to 10 passages^49^.

Nevertheless, an important question remains if ASCs from OI show bone regeneration capacity in vivo. In general, ASCs bone regeneration potential in combination with bioengineered scaffolds has been proven in various animal models^50^ with calvarial like-defect^51–54^, femoral head osteonecrosis^55^, femur defect^56^, distraction osteogenesis^57^, and spine fusion^58^. Additionally, ASCs bone regeneration potential has also been evaluated in case studies and small-size clinical trials in humans with cranial defects, cranio-maxillofacial skeleton defect, or osteoarthritis^59–62^. But it still needs to be evaluated if SVF from OI patients have the capability of bone regeneration in e.g., non-union fracture animal models or mouse models of OI. Mechanistically, we hypothesize that transplanted SVF will promote bone regeneration at the surgical site by "SVF-cells"-produced paracrine factors^63^ and by ASCs osteogenic differentiation ability itself. Furthermore, studies are also needed to evaluate the optimal delivery system of SVF to the desired site in OI. Encouraging results were recently published showing successful bone regeneration of undifferentiated temporomandibular joint synovial-fluid-MSCs from patients with temporomandibular dysfunctions on 3D polyetherketoneketone scaffolds in a rabbit calvarial critical-sized defect^64^.

Still, our study aimed to shed light on whether adipose tissue-derived SVF, taken from a pediatric OI-patient, can serve as autologous tissue/cell/bone graft to promote bone regeneration at the surgical site resulting in the prevention of a bone non-union in the same patient. It should be kept in mind that stem cells from OI patients contain mutations that cause OI; thus, the newly generated bone matrix will still be fragile. If the final therapy goal is to repair OI bones or produce a healthy bone matrix, stem cells from healthy individuals would be the better approach. Although this strategy has its drawbacks of (a) finding a donor (best: age- and sex-matched), (b) painful bone marrow harvest from the donor, and (c) graft-versus-host reaction in the OI patient leading to rejection of the transplant and other yet unknown consequences. Additionally, obtaining SVF from normal individuals would entail going through numerous regulatory hurdles imposed by e.g., FDA and Health Canada.

In conclusion, our study demonstrated the feasibility of isolating SVF-containing ASC from adipose tissue of pediatric OI patients. We demonstrated that yields of isolated ASC from OI patients are comparable to ASCs from healthy controls. And we verified that isolated ASCs from OI patients express the same stem cell markers and possess multi-lineage differentiation capacity as controls. Most importantly, osteogenic differentiation potential was irrespective of OI mutation. Thus, as a platform for future therapeutic use, SVF-containing ASC can be isolated within the same surgery and immediately be used for bone regeneration in OI patients.

## Material and methods

If not indicated otherwise, Supplemental Table A.5 summarizes catalogue numbers and company names of all chemicals used in this study.

### Study population

From August 2017 to November 2018, adipose tissue samples were collected from patients undergoing corrective surgery at our institution. Samples were collected from patients in the age range of 2–22 years diagnosed with either OI or other non-OI disorders (healthy controls, HC). Ethical permission (McGill Research Ethics and Compliance Committee, ID#A02-M15-11A) and written informed consent from all patients or their legal guardians were obtained. This research study was performed in accordance with institutional guidelines and Helsinki Ethical Principles for Medical Research Involving Human Patients.

### SVF isolation

**Figure 5:**
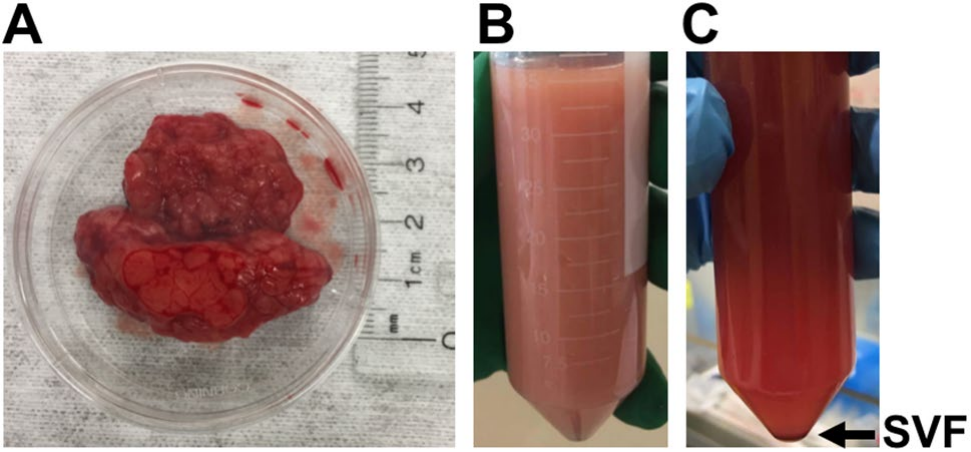
Schematic of the isolation of the stromal vascular fraction from adipose tissue. (**A**) Harvested adipose tissue. (**B**) Digested adipose tissue. (**C**) SVF obtained by differential centrifugation.

Isolation of SVF was performed according to the optimized method by Tevlin et al.^17^ with minor modifications. Briefly, harvested adipose tissue samples from the site of the surgical incision, were weighed and incubated in fetal bovine serum (FBS)-free ice cold culture medium (DMEM:F12 supplemented with 1% antibiotic–antimycotic and 1% penicillin–streptomycin) for 10 min at 4 °C. Then, adipose tissue samples were minced manually into small 1 × 1 mm pieces using sterile surgical scissors and homogenized using a 25 ml Sarstedt serological pipette. Subsequently, homogenized samples were mixed in a ratio of 1:2 (weight per volume, W/V; gram of adipose tissue/buffer) with freshly prepared digestion-collagenase buffer by dissolving 2.2 mg/ml collagenase (collagenase NB 6, GMP grade) in Hank's balanced salt solution supplemented with 10% bovine serum albumin (BSA) and incubated in a water bath at 37 °C for 60 min with vigorous shaking every 10 min for 1 min. Afterward, collagenase was neutralized by adding culture medium, followed by filtration using 100 µm membrane filters, and centrifugation at 700×*g* for 10 min. The pellet was collected as SVF (Fig. 5) and the number of nucleated cells counted using trypan blue. Finally, SVF was prepared for flow cytometry or cultured for multi-lineage differentiation.

### Flow cytometry

**Figure 6:**
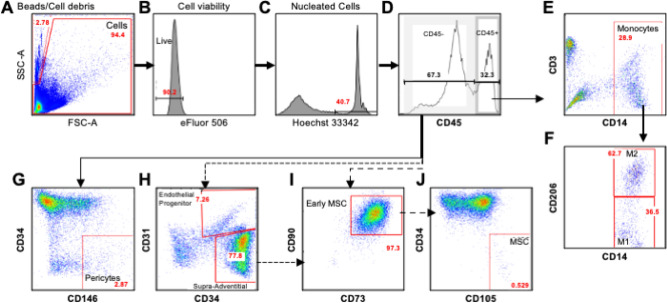
Gating strategy for the analysis of SVFs cell population by flow cytometry. SVF cell suspension was stained with different CD markers and dead cells were excluded by using Ebioscience eFluor 506 fixable viability dye labelling. (**A**) Forward and side scatterplot of viable cells (FSC-A axis; in figure 94.4% viable) and counting beads and cell debris (SSC-A axis; in Figure 2.78%). (**B**) Analysis of viable cells for live cells (in Figure 90.2%). (**C**) Analysis of viable cells for nucleated cells using Hoechst 33342 dye (in Figure 40.7%). (**D**) CD45 marker was used to distinguish between non-hematopoietic (in Figure 67.3%) and hematopoietic cell populations (in Figure 32.3%). (**E**) Hematopoietic population was further characterized by using CD14 and CD3 markers to separate monocytes/macrophage population (in Figure 28.9%), which was further characterized by using (**F**) CD14 and CD206 markers to individualize monocytes (M1, CD45^+^/CD14^+^/CD206^-^, in Figure 36.5%) and macrophages (M2, CD45^+^/CD14^+^/CD206^+^, in Figure 62.7%). Non-hematopoietic cell population was characterized by using (**G**) CD146 marker to identify pericytes (CD45^-^/CD34^-^/CD146^+^; 2.87%) and (**H**) CD31 marker to separate cells from endothelial origin (CD45^-^/CD34^+^/CD31^+^; 7.26%), and supra-adventitial stromal cells (CD45^-^/CD34^+^/CD31^-^; 77.8%). (**I**) Supra-adventitial stromal cells and non-hematopoietic cell population were pooled and further analyzed for the presence of early mesenchymal stem cells (CD45^-^/CD34^+^/CD73^+^/CD90^+^; in Figure 97.3%) using CD73 and CD90 markers. (**J**) Applying the marker CD105, early mesenchymal stem cells were further separated for the presence of activated mesenchymal stem cells (CD45^-^/CD34^+^/CD73^+^/CD90^+^/CD105^+^; in Figure 0.529%).

Characterization of the SVF cell subpopulation was carried out according to recommendations of the International Federation of Adipose Therapeutics and Science (IFATS) and the International Society for Cellular Therapy (ISCT). For flowcytometry, 2 × 10^6^ cells per ml diluted in ice cold blocking buffer (1X PBS, 0.5% BSA, 10 mM EDTA) were stained for 45 min at 4 °C in the dark with Hoechst 33342 trihydrochloride for nucleated cells, and the following fluorochrome-conjugated antibodies: CD34-APC, CD90-FITC, CD73-PE, CD31-APC-Cy7, CD146-PerCP-Cy5.5, CD45-PE-Cy7, CD105-PerCP-Cy5.5, CD14-PerCP-Cy5.5, CD206-APC, CD3-PE. To avoid emission overlapping, fluorochrome combinations were distributed into four separate independent measurements. Before signal acquisition viability dye eFluor 506 was added and signals were determined using Fortessa cell analyzer and analyzed using FlowJo software v.10.1. The following subpopulations were analysed: hematopoietic cells (CD45+), monocytes (CD45+/CD14+/CD206−), macrophages (CD45+/CD14+/CD206+), progenitor endothelial cells (CD45–/CD34+/CD31+), pericytes (CD45−/CD34−/CD146+), supra-adventitial stromal cells (CD45−/CD34+/CD31−), and early mesenchymal stem cells (CD45−/CD34+/CD73+/CD90+). Additionally, it was shown that activated ASCs increase the expression of CD105^23^, we analyzed the presence of “activated” mesenchymal stem cells (CD45−/CD34+/CD73+/CD90+/CD105+). Applied gating strategy is depicted in Fig. 6.

### Multi-lineage differentiation

Directly after isolation, SVF was seeded and cultured in culture medium in T25-culture flasks and non-adherent cells were removed the next day. Cells were harvested by TrypLE Express Enzyme at 80% confluence and then seeded at a density of 3000 cells/cm^2^ in T75-culture flasks. The medium was replaced every 3 days. Cells of passages 2 to 4 were used for multi-lineage differentiation.

For multi-lineage differentiation, cells were seeded at a density of 3000 cells/cm^2^ in 6-well plates for histochemical analysis and 12-well plates for RNA extraction. At 80% confluence, culture medium was replaced by osteogenic, adipogenic, or chondrogenic maintenance medium (referred as ‘treated’) or grown again in culture medium as control cells (referred as ‘non-treated’). For osteogenic and adipogenic differentiation, cells were exposed to induction media for 3 days, followed by maintenance media until the end of the experiment (Table 3). For chondrogenic differentiation, cells were exposed to chondrogenic induction medium during the entire experiment (Table 3). Media were replaced every 3 days for 21 days.

**Table 3:** Multi-lineage cell culture media.

Media	Composition
Osteogenic induction media	DMEM: F12
	10% FBS
	1% penicillin–streptomycin
	1% antibiotic–antimycotic
	10 nM dexamethasone
	10 mM β-glycerol phosphate
	50 µg/ml ascorbic-acid-2-phospahate
Osteogenic maintenance media	DMEM: F12
	10% FBS
	1% penicillin–streptomycin
	1% antibiotic–antimycotic
	10 nM dexamethasone
	5 mM β-glycerol phosphate
	50 µg/ml ascorbic-acid-2-phospahate
Adipogenic induction media	DMEM: F12
	10% FBS
	1% penicillin–streptomycin
	1% antibiotic–antimycotic
	10 µg/ml insulin
	1 µM dexamethasone
	500 µM 3-isobutyl-1-methylxanthine
Adipogenic maintenance media	DMEM: F12
	10% FBS
	1% penicillin–streptomycin
	1% antibiotic–antimycotic
	10 µg/ml insulin
Chondrogenic induction media	DMEM: F12
	10% FBS
	1% penicillin–streptomycin
	1% antibiotic–antimycotic
	10 nM dexamethasone
	50 µg/ml ascorbic-acid-2-phospahate
	10 ng/ml recombinant human TGF-β3
	40 μg/ml l-proline
	50 μg/ml ITS liquid media supplement

### Assessment of multi-lineage differentiation by histochemistry

On day 21 of culture, cells were fixed with either 70% ethanol for 60 min at − 20 °C for Alizarin Red staining or with 4% formalin for 60 min at room temperature for Oil Red or Alcian blue staining. Alizarin Red, Oil Red, and Alcian blue staining were performed at room temperature according to manufacturers' instructions. Briefly, Alizarin Red staining was performed for 10 min, followed by washes with distilled water and PBS; Oil Red staining was performed for 15 min; and Alcian blue staining for 120 min. Pictures were taken with LEICA DMRB microscope equipped with an Olympus DP70 digital camera, 10 ×/0.30 PL FLUOTAR objective or 40 ×/0.70 PL FLUOTAR objective, and the DP controller software.

### Alizarin red quantification

For quantification of deposited minerals, alizarin red stained cells were washed with deionized water and stain dissolved in 10% acetic acid. Absorbance was measured at 405 nm using a microplate reader (VICTOR Nivo).

### RNA extraction and real-time qPCR analyses

On day 7 and 21, total RNA was isolated using TRIzol™ reagent according to the manufacturer’s protocol. Reverse transcription of 500 ng RNA was performed using the High-Capacity cDNA Reverse Transcription Kit in the presence of RNase inhibitor. Real-time qPCR was performed with 25 ng of cDNA using an ABI 7500 Real-Time PCR Machine, TaqMan™ Universal PCR Master Mix, and the following human FAM labelled TaqMan® gene expression primers: Sox9 (Hs00165814-m1), OCN (Hs01587814-g1), Runx2 (Hs01047973-m1), ALPL (Hs01029144-m1), PPARg (Hs01115513-m1), Leptin (Ha00174877-m1), LEPR (Hs00174497-m1), Sox7 (Hs00846731-s1), ACAN (Hs00153936-m1), and Col10 (Hs00166657-m1) (all purchased from Applied Biosystems). Beta-actin (Hs01060665-g1) was used as endogenous control. Gene expression was analyzed according to the delta-delta Ct method and are presented as fold-changes (2^−delta-delta Ct^) of treated samples to non-treated samples.

### Statistical analyses

All isolated adipose tissue samples underwent same workflow of SVF isolation followed by separating the resulting SVF into one sample for flow cytometry analysis and another sample for in vitro multi-differentiation experiments. Due to technical issues such as insufficient number of isolated cells for flow cytometry analysis and multi-differentiation or contamination of cell cultures, the number of analysed samples varies. Thus, unless stated otherwise, data are presented as mean ± SEM (minimum—maximum). Data were evaluated for normal distribution using Shapiro–Wilk test. Non-normal distributed data were evaluated either using Mann -Whitney-Wilcoxon U-Test for 2 independent parameters such as HC vs OI, sex, and anatomical site, following established critical values of the smallest rank sum test^65^. Kruskal–Wallis-H test was used for evaluation of statistical difference for > 2 independent parameters like age-dependency, type of OI, and disease-causing collagen mutation. Normally distributed data were evaluated using independent T-test for 2 independent samples or one-way ANOVA for > 2 independent samples. Calculations were performed using SPSS software (v 24.0; SPSS Inc).

Statistical differences in gene expression between treated and non-treated samples were analyzed by paired T-test. Significant differences in genetic fold-change of OI versus HC were assessed by unpaired T-test. Calculations were performed using GraphPad Prism (v 8.1.1; GraphPad Software, www.graphpad.com) and p < 0.05 was considered significant.

## Supplementary Information

Supplementary Tables.
