# Supplementary material for: Characterization and functional analysis of the adipose tissue-derived stromal vascular fraction of pediatric patients with osteogenesis imperfecta
Source: Sci Rep. 2022 Feb 14;12:2414. doi: 10.1038/s41598-022-06063-4 (PMC8844034; doi:10.1038/s41598-022-06063-4)
Supplement: Supplementary file 1 — Supplementary Tables. [file 41598_2022_6063_MOESM1_ESM.doc]

**Supplement**

**Table A.1. Demographics of Study Cohort**

HC, healthy control; R, right side; L, left side.

**Table A.2. Statistical Analysis of Harvested Adipose Tissue Weight and Isolated Number of Cells of OI and HC Patients.**

Data are given in mean (SEM). Shapiro-Wilk test indicated that all data collected for each parameter are not distributed normally. Therefore, parameters with 2 independent samples were evaluated using the Mann-Whitney-Wilcoxon U-Test and parameters with >2 independent samples were evaluated using Kruskal-Wallis-H Test. NA, not applicable; $, *SERPINF1*, *IFITM5*, *WNT1*

**Table A.3. Statistical Analysis of SVF Composition of OI and HC Patients.**

NA, not applicable; $, *SERPINF1*, *IFITM5*, *WNT1*.

Data are given in mean (SEM). Analysis of normal distribution using Shapiro-Wilk test indicated that cell viability data are not normally distributed while data collected for early MSC, supra-adventitial stromal cells, and hematopoietic lineage are normally distributed. Cell viability data were evaluated using Mann-Whitney-Wilcoxon U-Test for parameters with 2 independent samples and Kruskal-Wallis-H Test for parameters with >2 independent samples. Early mesenchymal stem cells, supra-adventitial stromal cells, and hematopoietic lineage data were evaluated using independent t-test for 2 independent samples or one-way ANOVA for >2 independent samples.

**Table A.4. Continuing Statistical Analysis of SVF Composition of OI and HC Patients**

NA, not applicable; $, *SERPINF1*, *IFITM5*, *WNT1*.

Data are given as the mean (SEM). Analysis of normal distribution using Shapiro-Wilk test indicated that all data are not normally distributed. Data were evaluated using Mann-Whitney-Wilcoxon U-Test for parameters with 2 independent samples and Kruskal-Wallis-H Test for parameters with >2 independent samples.

**Table A.5. Resource Table**

**Supplement**

**Table A.1**. Demographics of Study Cohort

| **ID** | **Sex** | **Age**  **(years)** | **Diagnosis** | **Mutated gene** | **Anatomical site of the harvested adipose tissue** | **Adipose tissue weight (g)** | **Total viable cell yield (x10^6^)** | **Cells per gram of adipose tissue (x10^4^)** |
| --- | --- | --- | --- | --- | --- | --- | --- | --- |
| 1 | F | 10 | HC |  | Tibia L | 2.5 | 0.75 | 30 |
| 2 | F | 4 | HC |  | Hip R | 3.4 | 1.4 | 40 |
| 3 | M | 14 | HC |  | Tibia R | 5.2 | 5.0 | 96 |
| 4 | F | 12 | HC |  | Tibia R | 18.8 | 2.1 | 19 |
| 5 | M | 3 | OI type IV | *COL1A2* | Femur R | 1.76 | 0.56 | 32 |
| 6 | F | 6 | OI type IV | *COL1A1* | Tibia R | 3.6 | 2 | 55 |
| 7 | F | 6 | OI type IV | *COL1A1* | Femur R | 7.7 | 6 | 78 |
| 8 | F | 5 | OI type IV | *COL1A1* | Femur R | 4 | 0.67 | 17 |
| 9 | F | 2 | OI type IV | *COL1A1* | Femur and Tibia L | 2.6 | 3.3 | 125 |
| 10 | F | 6 | OI type IV | *COL1A1* | Tibia L | 5.8 | 2.6 | 45 |
| 11 | M | 14 | OI type IV | *COL1A1* | Femur and Tibia R | 8.6 | 8.1 | 95 |
| 12 | M | 9 | OI type IV | *COL1A1* | Femur R | 15 | 9.0 | 58 |
| 13 | F | 5 | OI type III | *COL1A2* | Tibia R | 4.7 | 4.1 | 88 |
| 14 | F | 4 | OI type VII | *CRTAP* | Femur R | 8.0 | 0.94 | 12 |
| 15 | M | 3 | OI type IV | *COL1A2* | Femur R | 3.4 | 2.7 | 80 |
| 16 | M | 13 | OI type VI | *SERPINF1* | Femur R | 7.3 | 1.6 | 22 |
| 17 | M | 21 | OI type IV | *COL1A2* | Tibia R | 1.2 | 1.4 | 117 |
| 18 | M | 21 | OI type IV | *COL1A2* | Femur R | 2.3 | 1.5 | 66 |
| 19 | M | 22 | OI type IV | *COL1A2* | Femur and Tibia L | 4.4 | 2.7 | 62 |
| 20 | F | 3 | OI type I | *COL1A1* | Femur R | 6.6 | 2.3 | 35 |
| 21 | F | 2 | OI type IV | *WNT1* | Femur R | 2.0 | 3.5 | 175 |
| 22 | M | 5 | OI type III | *COL1A2* | Tibia R | 2.6 | 1.7 | 67 |
| 23 | M | 5 | OI type III | *COL1A2* | Femur R | 6.2 | 1.9 | 32 |
| 24 | M | 10 | OI type IV | *COL1A1* | Femur L | 29 | 2.0 | 6.9 |
| 25 | M | 5 | OI type III | *COL1A2* | Femur and Tibia L | 6.4 | 2.5 | 42 |
| 26 | M | 9 | OI type IV | *COL1A2* | Femur R | 4.4 | 1.3 | 28 |
| 27 | M | 8 | OI type III | *COL1A2* | Tibia R | 8.4 | 1.8 | 21 |
| 28 | M | 5 | OI type IV | *COL1A2* | Femur R | 7.0 | 1.4 | 20 |
| 29 | F | 2 | OI type IV | *COL1A1* | Femur R | 5.5 | 1.4 | 25 |
| 30 | F | 8 | OI type IV | *COL1A1* | Femur R | 36 | 10 | 28 |
| 31 | M | 5 | OI type VII | *CRTAP* | Tibia R | 2.0 | 0.25 | 1.3 |
| 32 | F | 5 | OI type VII | *CRTAP* | Femur R | 13 | 1.4 | 13.8 |
| 33 | M | 10 | OI type III | *COL1A1* | Tibia R and L | 4.7 | 0.5 | 10 |
| 34 | F | 5 | OI type III | *COL1A2* | Femur R | 6.8 | 5.5 | 80.8 |
| 35 | M | 4 | OI type IV | *COL1A2* | Tibia L | 2.6 | 4.9 | 10.8 |
| 36 | F | 16 | OI type III | *COL1A2* | Femur R | 4.5 | 3.4 | 7.6 |
| 37 | F | 16 | OI type III | *COL1A2* | Femur R | 4.5 | 3.7 | 8.2 |
| 38 | F | 16 | OI type III | *COL1A2* | Femur R | 4.5 | 2.5 | 5.5 |
| 39 | F | 14 | OI type V | *IFITM5* | Tibia L | 5.0 | 0.63 | 12.5 |
| 40 | F | 14 | OI type V | *IFITM5* | Tibia L | 5.0 | 0.75 | 15.0 |

HC, healthy control; R, right side; L, left side.

**Table A.2: Statistical Analysis of Harvested Adipose Tissue Weight and Isolated Number of Cells of OI and HC Patients**

|  |  | **Adipose tissue weight (g)** | | **Total viable cell yield (x10^6^)** | | **Number of cells per**  **gram of adipose tissue (x10^4^)** | |
| --- | --- | --- | --- | --- | --- | --- | --- |
|  | **N** | **Mean (SEM)** | **P–value** | **Mean (SEM)** | **P–value** | **Mean (SEM)** | **P–value** |
| **HC vs OI** | | | | | | | |
| All | 40 | 6.93 (1.10) | 0.84 | 2.74 (0.36) | 0.80 | 44.56 (6.17) | 0.59 |
| HC | 4 | 7.48 (3.82) |  | 2.31 (0.94) |  | 46.25 (17.13) |  |
| all OI | 36 | 6.86 (1.17) |  | 2.79 (0.39) |  | 44.37 (6.66) |  |
| **Male vs female** | | | | | | | |
| All | 40 | 6.93 (1.1) | 0.57 | 2.74 (0.36) | 0.59 | 44.56 (6.17) | 0.57 |
| Male | 19 | 6.45 (1.46) |  | 2.67 (0.56) |  | 45.6 (7.8) |  |
| Female | 21 | 7.36 (1.65) |  | 2.8 (0.49) |  | 43.6 (9.6) |  |
| **Age–dependency** | | | | | | | |
| All | 40 | 6.93 (1.1) | 0.10 | 2.74 (0.36) | 0.75 | 44.56 (6.17) | 0.09 |
| Prepubertal  (1–12 years) | 29 | 7.74 (1.48) |  | 2.7 (0.44) |  | 43.98 (7.16) |  |
| Pubertal  (13–16 years) | 8 | 5.58 (0.54) |  | 3.21 (0.88) |  | 32.72 (13.82) |  |
| Postpubertal  (>16 years) | 3 | 2.63 (0.94) |  | 1.87 (0.42) |  | 81.67 (17.7) |  |
| **Type of OI** | | | | | | | |
| All | 36 | 6.86 (1.17) | 0.82 | 2.79 (0.39) | 0.10 | 44.37 (6.66) | 0.13 |
| OI–IV | 19 | 7.73 (2.15) |  | 3.42 (0.66) |  | 59.14 (10.21) |  |
| OI–III | 10 | 5.33 (0.51) |  | 2.76 (0.46) |  | 36.21 (10.08) |  |
| OI–I | 1 | N/A |  | N/A |  | N/A |  |
| OI–V | 2 | 5 (0) |  | 0.69 (0.06) |  | 13.75 (1.25) |  |
| OI–VI | 1 | N/A |  | N/A |  | N/A |  |
| OI–VII | 3 | 7.67 (3.18) |  | 0.86 (0.33) |  | 9 (3.91) |  |
| **Disease causing gene mutation** | | | | | | | |
| All | 36 | 6.86 (1.17) | 0.09 | 2.79 (0.39) | 0.05 | 44.37 (6.66) | 0.28 |
| *COL1A1* | 12 | 10.76 (3.1) |  | 3.99 (0.97) |  | 48.16 (10.44) |  |
| *COL1A2* | 17 | 4.45 (0.49) |  | 2.56 (0.33) |  | 45.17 (8.15) |  |
| Others ^$^ | 7 | 6.04 (1.45) |  | 1.29 (0.41) |  | 35.93 (23.29) |  |
| **Anatomical side: left vs right** | | | | | | | |
| All | 40 | 6.93 (1.1) | 0.96 | 2.74 (0.36) | 0.64 | 44.56 (6.17) | 0.42 |
| Left | 10 | 7.63 (2.15) |  | 2.15 (0.42) |  | 36.3 (11.4) |  |
| Right | 30 | 6.69 (1.22) |  | 2.94 (0.46) |  | 47.3 (7.3) |  |
| **Anatomical side: femur vs tibia** | | | | | | | |
| All | 34 | 6.93 (1.1) | 0.16 | 2.74 (0.36) | 0.45 | 44.56 (6.17) | 0.66 |
| Femur | 21 | 8.55 (1.89) |  | 3 (0.57) |  | 39.56 (8.68) |  |
| Tibia | 13 | 5.19 (1.25) |  | 2.16 (0.44) |  | 44.43 (10.36) |  |

Data are given in mean (SEM). Shapiro-Wilk test indicated that all data collected for each parameter are not distributed normally. Therefore, parameters with 2 independent samples were evaluated using the Mann-Whitney-Wilcoxon U-Test and parameters with >2 independent samples were evaluated using Kruskal-Wallis-H Test. NA, not applicable; $, *SERPINF1*, *IFITM5*, *WNT1*

**Table A.3. Statistical analysis of SVF composition of OI and HC patients**

|  | **Cell viability** | | | **N** | **Early mesenchymal stem cells**  (CD45–/CD34+/CD73+/CD90+) | | **Supra-adventitial stromal cells**  (CD45– CD34+ CD31–) | | **Hematopoietic lineage**  (CD45+) | |  |
| --- | --- | --- | --- | --- | --- | --- | --- | --- | --- | --- | --- |
|  | **N** | **Mean (SEM)** | **P–value** |  | **Mean (SEM)** | **P–value** | **Mean (SEM)** | **P–value** | **Mean (SEM)** | **P–value** |  |
| **HC vs OI** | | | | | | | | | | | |
| All | 18 | 84.24 (3.42) | 0.31 | 21 | 35.54 (4.49) | 0.95 | 38.56 (4.7) | 0.79 | 15.98 (1.56) | 0.72 |  |
| HC | 3 | 80.53 (5.33) |  | 3 | 34.82 (2.84) |  | 41.77 (4.99) |  | 17.39 (2.52) |  |  |
| OI | 15 | 84.98 (4) |  | 18 | 35.66 (5.24) |  | 38.02 (5.45) |  | 15.75 (1.78) |  |  |
| **Female vs male** | | | | | | | | | | | |
| All | 18 | 84.24 (3.42) | 0.07 | 21 | 35.54 (4.49) | **0.02** | 38.56 (4.7) | **0.02** | 15.98 (1.56) | 0.67 |  |
| Male | 9 | 77.99 (5.66) |  | 9 | 47.27 (6.09) |  | 50.79 (5.94) |  | 15.19 (2.22) |  |  |
| Female | 9 | 90.49 (2.82) |  | 12 | 26.74 (5.24) |  | 29.39 (5.75) |  | 16.58 (2.22) |  |  |
| **Age–dependency** | | | | | | | | | | | |
| All | 18 | 84.24 (3.42) | 0.06 | 21 | 35.54 (4.49) | **0.015** | 38.56 (4.7) | **0.023** | 15.98 (1.56) | 0.69 |  |
| Prepubertal  (1–12 years) | 9 | 86.63 (3.21) |  | 12 | 35.12 (4.86) |  | 38.19 (5.29) |  | 15.3 (2.47) |  |  |
| Pubertal  (13–16 years) | 6 | 90.56 (4.5) |  | 6 | 22.9 (5.88) |  | 25.8 (6.43) |  | 15.64 (0.79) |  |  |
| Postpubertal  (>16 years) | 3 | 64.45 (11.34) |  | 3 | 62.5 (13.92) |  | 65.56 (13.94) |  | 19.39 (5.08) |  |  |
| **Type of OI** | | | | | | | | | | | |
| All | 15 | 84.24 (3.42) | **0.011** | 18 | 35.54 (4.49) | 0.42 | 38.56 (4.7) | 0.43 | 15.98 (1.56) | 0.68 |  |
| OI–IV | 7 | 96.65 (0.52) |  | 6 | 22.78 (5.11) |  | 25.19 (5.45) |  | 15.47 (2.39) |  |  |
| OI–III | 6 | 73.59 (5.91) |  | 9 | 44.75 (8.79) |  | 47.84 (9.12) |  | 14.55 (3.02) |  |  |
| OI–V | 1 | N/A |  | 1 | N/A |  | N/A |  | N/A |  |  |
| OI–VI | 1 | N/A |  | 1 | N/A |  | N/A |  | N/A |  |  |
| OI–VII | 0 | N/A |  | 1 | N/A |  | N/A |  | N/A |  |  |
| **Disease causing genetic mutation** | | | | | | | | | | | |
| All | 15 | 84.98 (4) | 0.97 | 18 | 35.66 (5.24) | 0.44 | 38.02 (5.45) | 0.49 | 15.75 (1.78) | 0.21 |  |
| *COL1A1* | 1 | N/A |  | 3 | 20.18 (15.19) |  | 22.97 (16.93) |  | 9.43 (7.27) |  |  |
| *COL1A2* | 11 | 84.9 (5.31) |  | 11 | 38.27 (7.02) |  | 40.91 (7.16) |  | 16.03 (1.88) |  |  |
| Others ^$^ | 3 | 84.98 (6.61) |  | 4 | 40.08 (7.81) |  | 41.38 (8.68) |  | 19.7 (2.63) |  |  |
| **Anatomical side: left vs right** | | | | | | | | | | | |
| All | 18 | 84.24 (3.42) | 0.68 | 21 | 35.54 (4.49) | 0.81 | 38.56 (4.7) | 0.94 | 15.98 (1.56) | 0.14 |  |
| Left | 3 | 86.92 (5.45) |  | 3 | 32.72 (5.96) |  | 37.64 (7.68) |  | 21.69 (4.33) |  |  |
| Right | 15 | 83.7 (4.01) |  | 18 | 36.01 (5.18) |  | 38.71 (5.39) |  | 15.03 (1.61) |  |  |
| **Anatomical side: tibia vs femur** | | | | | | | | | | | |
| All | 16 | 83.91 (3.85) | 1.0 | 19 | 35.73 (4.96) | 0.91 | 38.82 (5.19) | 0.79 | 15.18 (1.55) | 0.74 |  |
| Femur | 10 | 85.79 (3.82) |  | 12 | 35.26 (5.88) |  | 37.74 (6.26) |  | 15.59 (1.94) |  |  |
| Tibia | 6 | 80.78 (8.45) |  | 7 | 36.54 (9.57) |  | 40.67 (9.78) |  | 14.49 (2.78) |  |  |

NA, not applicable; $, *SERPINF1*, *IFITM5*, *WNT1*.

Data are given in mean (SEM). Analysis of normal distribution using Shapiro-Wilk test indicated that cell viability data are not normally distributed while data collected for early MSC, supra-adventitial stromal cells, and hematopoietic lineage are normally distributed. Cell viability data were evaluated using Mann-Whitney-Wilcoxon U-Test for parameters with 2 independent samples and Kruskal-Wallis-H Test for parameters with >2 independent samples. Early mesenchymal stem cells, supra-adventitial stromal cells, and hematopoietic lineage data were evaluated using independent t-test for 2 independent samples or one-way ANOVA for >2 independent samples.

**Table A.4. Continuing Statistical Analysis of SVF Composition of OI and HC Patients**

|  | **N** | **Activated mesenchymal stem cells**  (CD45–/CD34+/CD73+/CD90+/CD105+) | | **Progenitor endothelial cells**  (CD45– CD34+ CD31+) | | **Pericytes**  (CD45–/CD34–/CD146+) | | **Monocytes**  (CD45+/CD14+/CD206–) | | **Macrophages**  (CD45+/CD14+/CD206+) | |
| --- | --- | --- | --- | --- | --- | --- | --- | --- | --- | --- | --- |
|  |  | **Mean (SEM)** | **P–value** | **Mean (SEM)** | **P–value** | **Mean (SEM)** | **P–value** | **Mean (SEM)** | **P–value** | **Mean (SEM)** | **P–value** |
| **HC vs OI** | | | | | | | | | | | |
| All | 21 | 0.04 (0.02) | 0.46 | 5.99 (1.46) | **0.035** | 3.92 (1.28) | 0.37 | 2.86 (0.77) | 0.55 | 3.97 (0.85) | 0.69 |
| HC | 3 | 0 (0) |  | 12.6 (4.92) |  | 4.02 (1.65) |  | 3.61 (2.24) |  | 2.24 (0.33) |  |
| OI | 18 | 0.05 (0.03) |  | 4.89 (1.4) |  | 3.91 (1.48) |  | 2.74 (0.84) |  | 4.26 (0.98) |  |
| **Female vs male** | | | | | | | | | | | |
| All | 21 | 0.04 (0.02) | 0.12 | 5.99 (1.46) | **0.007** | 3.92 (1.28) | 0.67 | 2.86 (0.77) | **0.04** | 3.97 (0.85) | 0.32 |
| Male | 9 | 0 (0) |  | 10.12 (2.69) |  | 4.55 (2.56) |  | 4.25 (1.28) |  | 4.8 (1.56) |  |
| Female | 12 | 0.07 (0.04) |  | 2.9 (0.9) |  | 3.45 (1.26) |  | 1.83 (0.88) |  | 3.35 (0.94) |  |
| **Age–dependency** | | | | | | | | | | | |
| All | 21 | 0.04 (0.02) | 0.29 | 5.99 (1.46) | 0.31 | 3.92 (1.28) | **0.014** | 2.86 (0.77) | 0.08 | 3.97 (0.85) | 0.20 |
| Prepubertal  (1–12 years) | 12 | 0.07 (0.04) |  | 4.83 (1.65) |  | 5.95 (2.07) |  | 2.33 (0.89) |  | 3.61 (0.9) |  |
| Pubertal  (13–16 years) | 6 | 0 (0) |  | 5.74 (3.23) |  | 0.67 (0.3) |  | 1.86 (1.25) |  | 3.38 (2.03) |  |
| Postpubertal  (>16 years) | 3 | 0 (0) |  | 11.13 (4.6) |  | 2.32 (0.35) |  | 7 (2.5) |  | 6.61 (2.81) |  |
| **Type of OI** | | | | | | | | | | | |
| All | 21 | 0.04 (0.02) | 1.0 | 5.99 (1.46) | **0.048** | 3.92 (1.28) | 0.11 | 2.86 (0.77) | 0.07 | 3.97 (0.85) | 0.21 |
| OI–IV | 6 | 0 (0) |  | 2.34 (0.37) |  | 2.8 (2.43) |  | 0.72 (0.5) |  | 2.69 (0.87) |  |
| OI–III | 9 | 0.05 (0.04) |  | 7.25 (2.56) |  | 5.39 (2.5) |  | 3.47 (1.24) |  | 3.95 (1.32) |  |
| OI–VII | 1 | NA |  | NA |  | N/A |  | N/A |  | N/A |  |
| OI–V | 1 | NA |  | NA |  | N/A |  | N/A |  | N/A |  |
| OI–VI | 1 | NA |  | NA |  | N/A |  | N/A |  | N/A |  |
| **Disease causing genetic mutation** | | | | | | | | | | | |
| All | 18 | 0.05 (0.03) | **0.02** | 4.89 (1.4) | 0.48 | 3.91 (1.48) | 0.95 | 2.74 (0.84) | 0.37 | 4.26 (0.98) | 0.24 |
| *COL1A1* | 3 | 0.16 (0.1) |  | 1.85 (1.01) |  | 3.05 (1.97) |  | 0.76 (0.65) |  | 3.1 (2.54) |  |
| *COL1A2* | 11 | 0 (0) |  | 6.43 (2.14) |  | 4.84 (2.37) |  | 2.87 (1.1) |  | 3.48 (1.02) |  |
| Others ^$^ | 4 | 0.08 (0.08) |  | 2.94 (1.25) |  | 1.97 (0.55) |  | 3.87 (2.33) |  | 7.28 (2.76) |  |
| **Anatomical side: left vs right** | | | | | | | | | | | |
| All | 21 | 0.04 (0.02) | 0.46 | 5.99 (1.46) | 0.42 | 3.92 (1.28) | 0.76 | 2.86 (0.77) | 0.42 | 3.97 (0.85) | 0.55 |
| Left | 3 | 0 (0) |  | 8.16 (3.25) |  | 3.36 (1.9) |  | 3.44 (1.82) |  | 5.48 (3.4) |  |
| Right | 18 | 0.05 (0.03) |  | 5.63 (1.63) |  | 4.02 (1.47) |  | 2.77 (0.86) |  | 3.72 (0.86) |  |
| **Anatomical side: femur vs tibia** | | | | | | | | | | | |
| All | 19 | 0.04 (0.04) | 1.0 | 5.82 (1.59) | 0.67 | 3.98 (1.42) | 0.80 | 2.79 (0.81) | 0.5 | 3.63 (0.82) | 0.72 |
| Femur | 12 | 0.04 (0.03) |  | 5.48 (1.98) |  | 4.1 (1.96) |  | 2.93 (1.18) |  | 4.16 (1.22) |  |
| Tibia | 7 | 0.05 (0.05) |  | 6.41 (2.88) |  | 3.78 (2.05) |  | 2.54 (1) |  | 2.74 (0.76) |  |

NA, not applicable; $, *SERPINF1*, *IFITM5*, *WNT1*.

Data are given as the mean (SEM). Analysis of normal distribution using Shapiro-Wilk test indicated that all data are not normally distributed. Data were evaluated using Mann-Whitney-Wilcoxon U-Test for parameters with 2 independent samples and Kruskal-Wallis-H Test for parameters with >2 independent samples.

**Table A.5.** Resource Table

| **Reagent** | **Source** | **Catalog number** |
| --- | --- | --- |
| **Chemicals and reagents** |  |  |
| TRIzol Reagent | Ambion | 15596018 |
| High Capacity cDNA Reverse Transcription Kit | Applied Biosystems | 4368813 |
| RNase Inhibitor | Applied Biosystems | N8080119 |
| TaqMan Universal PCR Master Mix | Applied Biosystems | 4364340 |
| TrypLE Express Enzyme | Gibco | 12604012 |
| Bovine serum albumin (BSA) | Sigma-Aldrich | A7030-50G |
| EDTA | Fisher Chemical | S311-500 |
| Trypan blue 0.4% | Gibco | T8154-100ML |
| Fetal bovine serum (FBS) | Gibco | 12483020 |
| Low glucose Dulbecco’s Modified Eagle Medium Nutrient Mixture F-12(Ham) (DMEM:F12) | Gibco | 11330032 |
| Collagenase NB 6, GMP grade | SERVA | 17458.03 |
| HANK’S balanced salt solution (HBSS) | Sigma-Aldrich | H6648-1L |
| PBS pH7.4 (1x) | Gibco | 10010023 |
| Penicillin-streptomycin | Gibco | 15140122 |
| Antibiotic-antimycotic | Gibco | 15240062 |
| 10% Buffered Formalin Acetate | Fisher Chemical | SF99-4 |
| Ethanol | Greenfield Global | P016EAAN |
| Dexamethasone | Sigma-Aldrich | D4902-100MG |
| β-glycerol phosphate | Sigma-Aldrich | G9422-50G |
| Ascorbic-acid-2-phospahate | Sigma-Aldrich | A8960-5G |
| Insulin | Sigma-Aldrich | I2643-0MG |
| 3-isobutyl-1-methylxanthine | Sigma-Aldrich | I5879-1G |
| Recombinant human transforming growth factor-beta 3 (TGF-β3) | Prospec | CYT-368 |
| L-Proline | Sigma-Aldrich | P5607-25G |
| ITS liquid media supplement | Sigma-Aldrich | I3146-5ML |
| Acetic Acid | EMD | AX0079-2 |
|  |  |  |
| **Antibodies** |  |  |
| CD34-APC | BD Pharmingen Inc | 555824 |
| CD90-FITC | BD Pharmingen Inc | 555595 |
| CD73-PE | BD Pharmingen Inc | 550257 |
| CD31-APC-Cy7 | BD Pharmingen Inc | 563653 |
| CD146-PerCP-Cy5.5 | BD Pharmingen Inc | 562134 |
| CD45-PE-Cy7 | BD Pharmingen Inc | 560915 |
| CD105-PerCP-Cy5.5 | BD Pharmingen Inc | 560819 |
| CD14-PerCP-Cy5.5 | BD Pharmingen Inc | 562692 |
| CD206-APC | BD Pharmingen Inc | 561763 |
| CD3-PE | BD Pharmingen Inc | 555333 |
| CountBright absolute counting beads | Thermo Fisher Invitrogen | C36950 |
| Fixable Viability Stain eFluor506 | Thermo-Fisher | 65086614 |
| Hoechst 33342 trihydrochloride | Thermo-Fisher | H3570 |
|  |  |  |
| **Staining solutions** |  |  |
| Alizarin Red S stain | Sigma-Aldrich | A5533-25G |
| Oil Red staining | Sigma-Aldrich | O0625-25G |
| Alcian blue staining | Sigma-Aldrich | A5268-10G |
